# An Eco-Friendly Method for Saturated Hydrocarbon Chlorination: Exploring the Potential of First-Row Transition Metal Ions

**DOI:** 10.1021/acsomega.5c01390

**Published:** 2025-06-25

**Authors:** Eduardo S. Neves, Leonardo M. Lube, Lívia R. Oliveira, Christiane Fernandes, Adolfo Horn

**Affiliations:** a Laboratório de Ciências Químicas, Universidade Estadual do Norte Fluminense Darcy Ribeiro, Campos dos Goytacazes, RJ 28013-602, Brazil; b 119503 Instituto Federal Fluminense, Campus Campos Centro, Campos dos Goytacazes, RJ 28030-130, Brazil; c Departamento de Química, 28117 Universidade Federal de Santa Catarina, Florianópolis, SC 88040-900, Brazil

## Abstract

The functionalization of saturated hydrocarbons in a controlled, efficient, and selective manner represents a significant challenge in modern chemistry due to the low reactivity of their C–H bonds. This study introduces a new chlorination method using trichloroisocyanuric acid (TCCA) as the halogenating agent and first-row transition metal salts as catalysts. The investigation utilized four different substrates (cyclohexane, *n*-hexane, cycloheptane, and norbornane) along with eight transition metal ions (V­(IV), Cr­(III), Mn­(II), Fe­(II), Ni­(II), Co­(II), Cu­(II), and Zn­(II)) as potential catalysts and further Li^+^. The reactions were conducted with a substrate:TCCA:catalyst ratio of 1000:333:1 at 25, 50, and 75 °C for 24 h. The highest yields were obtained with Cu­(ClO_4_)_2_.6H_2_O at 75 °C: 42.1 ± 0.6% for chlorocyclohexane, 44.6 ± 0.7% for chlorocycloheptane, 42.1 ± 0.8% for exo-2-chloronorbornane, and 22.7 ± 0.6% for 2-chlorohexane. While higher temperatures (75 °C) enhance product yields, they also lower selectivity by promoting the formation of dichlorinated products. The reported system offers a safer and more efficient alternative for the selective chlorination of saturated hydrocarbons, achieving high yields and broad applicability.

## Graphical abstract

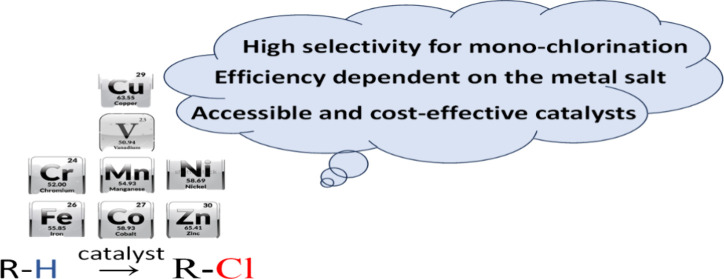

## 1 Introduction

Alkanes are abundant in nature, and in comparison with unsaturated hydrocarbons, alkenes and alkynes, show much lower reactivity.^1,2^ Such behavior occurs because the simple chemical bonds in alkanes show low polarization, as the electronegativity of their constituent atoms, carbon and hydrogen, is similar.^3–5^ Thus, developing processes to functionalize alkanes may yield more significant and valuable chemical compounds,^6,7^ making the selective functionalization of C–H bonds in alkanes a subject of considerable scientific and industrial interest.^8–11^ In this way, one of the greatest challenges in modern chemistry is the functionalization of saturated hydrocarbons with high yield and selectivity employing environmentally friendly processes, rendering oxygenated products as alcohols, ketones, aldehydes, and carboxylic acids.^12–15^ For instance, the cyclohexane functionalization is of industrial interest, as exemplified by the utilization of cyclohexanol and cyclohexanone in the polymer industry.^16–18^ Chlorocyclohexane is another example, being utilized as a precursor for the syntheses of N-cyclohexylthiophthalimide, trihexaphenidyl, and azocyclotin compounds used by the rubber, pharmaceutical, and pesticide industries, respectively.^7,19,20^

Concerning haloalkanes, alkyl chlorides are traditionally obtained through three methods: (a) substitution of the hydroxyl group in saturated aliphatic alcohols with hydrochloric acid,^21^ (b) addition of hydrochloric acid to alkenes,^22^ and (c) chlorination of alkanes using molecular chlorine.^23^ Methods (a) and (b) offer several advantages such as mild operating conditions and excellent selectivity for monochlorinated products. However, the high cost of raw materials (compared with hydrocarbons) makes these two methods less attractive. On the other hand, method (c) utilizes abundant and low-cost raw materials, providing high conversion efficiency. Nevertheless, it utilizes molecular chlorine, a strong oxidant that poses technical challenges due to its toxicity, corrosiveness, and potential for explosion, complicating its handling and operation. Furthermore, this reaction exhibits low selectivity for monochlorinated products, as the formation of polychlorinated products is almost inevitable. Additionally, it generates hydrochloric acid (a strong acid) as a byproduct, which does not align with the green chemistry principles.^19,23,24^

Regarding oxidant reagents for functionalization of hydrocarbons, *N*-haloamines, *N*-haloamides, *N*-halosaccharins, and *N*-halosulfonamides have gained importance in various organic syntheses (oxidation reactions,^25^ halogenation,^26–28^ epoxidation,^29^ and acylation^30^). Among these *N*-halo reagents, *N*-haloamides, particularly trihaloisocyanuric acids (Figure 1), hold a prominent position due to their high atom efficiency. Examples include trichloroisocyanuric acid (TCCA), tribromoisocyanuric acid (TBCA), and triiodoisocyanuric acid (TICA), which, respectively, can transfer up to 45.5, 65.5, and 75.1%, of their masses to the substrate.^31–33^

**Figure 1:**
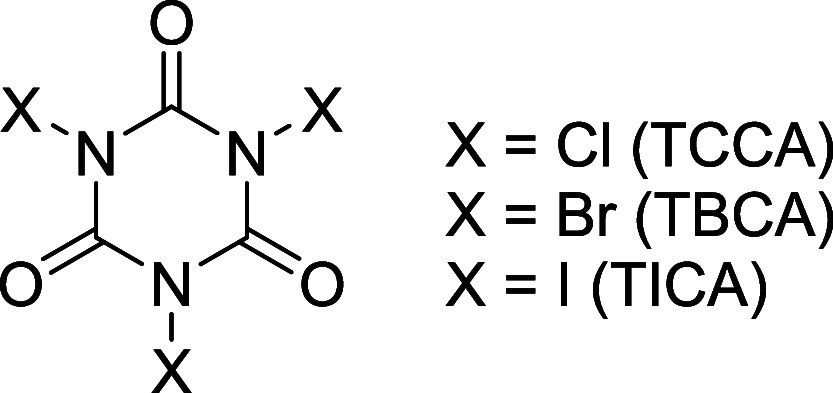
General structure of trihaloisocyanuric acids.

Specifically, trichloroisocyanuric acid (1,3,5-trichloro-1,3,5-triazine-2,4,6-(1*H*,3*H*,5*H*)-trione; TCCA) was first reported in 1902 by Chattaway and Wadmore.^34^ Its synthesis is achieved through the reaction between cyanuric acid and chlorine gas in an aqueous solution of sodium hydroxide.^34,35^

TCCA is recognized as an excellent oxidant and a chlorinating agent. It is a solid, easy to handle, stable, and low-cost reagent. It operates effectively under moderate reaction conditions, such as temperature and pressure, with an optimal stoichiometric molar ratio of the reagents. These qualities make TCCA particularly appealing for large-scale industrial applications.^33^ Another advantage of TCCA in organic reactions is that the byproduct (cyanuric acid) usually precipitates in organic solvents, making it easy to recover through filtration and reuse for producing more TCCA. This makes the process more cost-effective and generates minimal waste, aligning with the principles of green chemistry.^36^ Furthermore, it is proposed that in the presence of water, TCCA undergoes hydrolysis, generating hypochlorous acid (HClO), which can be activated by metal ions.^26–28,37^

Until 2017, TCCA was used mainly for the oxidation of functionalized hydrocarbons (alkenes, alkynes, arenes, etc.). Our group was the pioneer in showing that TCCA could halogenate alkanes in a process catalyzed by coordination compounds.^26–28,38^ Among the coordination compounds tested until now, the copper­(II) compound [Cu­(BPAH)­(H_2_O)]­(ClO_4_)_2_ (BPAH = 1,4-bis­(propanamide)­homopiperazine) has shown the highest activity.^27^ However, during our studies, we have observed that some common metal salts showed potential in performing a similar reaction. Therefore, we are reporting a comprehensive study on the effectiveness of the following transition metal salts in promoting the chlorination of cyclohexane, *n*-hexane, cycloheptane, and norbornane: VOSO_4_·5H_2_O, Cr­(ClO_4_)_3_·6H_2_O, Mn­(ClO_4_)_2_·6H_2_O, Fe­(ClO_4_)_2_·6H_2_O, Ni­(ClO_4_)_2_·6H_2_O, Co­(ClO_4_)_2_·6H_2_O, Cu­(ClO_4_)_2_·6H_2_O, CuCl_2_·2H_2_O, Cu­(NO_3_)_2_·3H_2_O, CuSO_4_·5H_2_O, Cu­(CH_3_COO)_2_·H_2_O, and Zn­(ClO_4_)_2_·6H_2_O). Our findings indicate that Cu­(ClO_4_)_2_·6H_2_O is a promising catalyst for the monohalogenation of saturated hydrocarbons.

## 2 Experimental Section

The halogenation reactions were performed as described previously,^26–28^ using 7 × 10^–4^ mol L^–1^ of catalyst, 7 × 10^–1^ mol L^–1^ of substrate, and 2.33 × 10^–1^ mol L^–1^ of TCCA, representing a ratio of 1:1000:333 equiv of catalyst:substrate:TCCA. It was carried out in acetonitrile, with a final volume of 5.0 mL, at 25, 50, or 75 °C for 24 h. A control reaction, without a catalyst in the reaction system, was prepared and analyzed to compare the yields obtained from reactions performed in the presence of the catalysts and evaluate the catalytic efficiency after 24 h of reaction. A 250 μL aliquot was withdrawn and diluted with 1250 μL of acetonitrile in a separate vial. Subsequently, 50 μL of the resulting solution were taken to which 100 μL of the internal standard solution (2-ethylhexanol; 7 × 10^–2^ mol L^–1^ in CH_3_CN) and 850 μL of acetonitrile were added. The total dilution was 1:120. All of the studies were performed in triplicate. The reaction product was quantified using GC-FID in a VARIAN gas chromatograph, model GC-450, equipped with a Varian CP WAX capillary column (30 m × 0.35 mm × 0.25 μm). The identity of the product was determined by mass spectrometry (GC-MS) on an AGILENT 7890A gas chromatograph equipped with an HP 5MS column (30 m × 0.25 mm × 0.25 μm). Nuclear magnetic resonance spectra (^1^H NMR) were recorded on a Bruker Ascend 500 AVANCE III HD spectrometer operating at 500 MHz for ^1^H or on a VARIAN-FT-NMR 400 MHz spectrometer operating at 400 MHz for ^1^H. For sample preparation, spectroscopic-grade acetonitrile was used, and a combination of CH_3_CN/D_2_O (90:10) was employed for equipment shimming. *N*,*N*-Dimethylformamide (DMF) was used as an internal standard and for chemical shift (δ, ppm) referencing. The analysis method involved using a 500 μL aliquot of the reaction medium combined with *N*,*N*-dimethylformamide (DMF) in a 1:1 molar ratio with the evaluated substrate. The chlorination products were quantified by comparing the integrated signal areas of the product’s hydrogens with those of the internal standard’s hydrogens.

The most effective catalytic system was selected and used to evaluate the chlorination of additional substrates, including *n*-hexane, cycloheptane, and norbornane. Product identification and quantification were performed by using GC-MS and ^1^H NMR, respectively. The reaction mixture consisted of the same ratio of 1:1000:333 equiv of catalyst:substrate:TCCA, in acetonitrile as the solvent, with a final volume of 2.0 mL. The reactions were conducted at 25, 50, and 75 °C for 24 h, as shown in Figure S1. The chromatograms and NMR spectra are shown as the Supporting Information (Figures S2–S17).

EPR studies were carried out using a Bruker EMX micro-9.5/2.7/P/L system using a highly sensitive cylindrical cavity operating in X-band (∼9.4 GHz) at 295 K, using the following experimental settings: central magnetic field of 3000 G, sweep width of 1800 G, microwave power of 5 mW, modulation amplitude of 4 G, modulation frequency of 100 kHz, receiver gain of 30 dB, sweep time of 20 s, and number of scans of 4. To investigate the signals associated with DMPO adducts, the modulation amplitude was set to 1 G. Stock solutions of the best catalyst, Cu­(ClO_4_)_2_·6H_2_O, and its less active counterpart, CuCl_2_·2H_2_O, were prepared in acetonitrile at a concentration of 10 mM. The spectra were measured using glass capillary tubes (BlauBrand). The EPR spectra of the pure compounds were obtained from a solution prepared by mixing 200 μL of the stock solution with 75 μL of acetonitrile. For the experiments conducted in the presence of TCCA, new solutions were prepared by combining 200 μL of the stock solution containing the copper salt of interest, 25 μL of acetonitrile, and 50 μL of a TCCA solution at a concentration of 100 μM, also prepared in acetonitrile. For the EPR analyses of the systems containing the copper salts, TCCA and DMPO, the solutions were prepared by mixing 200 μL of the copper salt stock solution, 25 μL of a 400 μM DMPO solution prepared in acetonitrile, and 50 μL of the TCCA stock solution. The spectra were registered 180 s after mixing and were conducted at 295 K. The simulated spectrum was calculated using SpinFit software supplied by the manufacturer.

## 3 Results and Discussion

### 3.1 Screening of the Catalytic Activity

Table 1 shows the results of the catalytic activity of diverse transition metal ions in the chlorination reaction of cyclohexane employing TCCA as the halogenating agent. The study revealed that the salt copper­(II) perchlorate hexahydrate exhibited the highest activity (Table 1, entry 7), reaching around 15% of conversion at 25 °C. In contrast, the activity of the other transition metal ions evaluated (VOSO_4_·5H_2_O, Cr­(ClO_4_)_3_·6H_2_O, Mn­(ClO_4_)_2_·6H_2_O, Fe­(ClO_4_)_2_·6H_2_O, Co­(ClO_4_)_2_·6H_2_O, Ni­(ClO_4_)_2_·6H_2_O, and Zn­(ClO_4_)_2_·6H_2_O) was significantly lower (0–6%).

**Table 1:** Yields Obtained in the Chlorination Reaction of Cyclohexane Using TCCA as an Oxidant and Transition Metal Salts as a Catalyst

entry	catalyst^a^	% yield of chlorocyclohexane^b^
1	VOSO_4_·5H_2_O	6.3 ± 0.3
2	Cr(ClO_4_)_3_·6H_2_O	3.9 ± 0.9
3	Mn(ClO_4_)_2_·6H_2_O	3.2 ± 0.2
4	Fe(ClO_4_)_2_·6H_2_O	1.6 ± 0.5
5	Co(ClO_4_)_2_·6H_2_O	2.4 ± 0.6
6	Ni(ClO_4_)_2_·6H_2_O	3.5 ± 0.4
7	Cu(ClO_4_)_2_·6H_2_O	15.9 ± 0.7
8	Cu(ClO_4_)_2_·6H_2_O^c^	16.5 ± 0.9
9	Cu(ClO_4_)_2_·6H_2_O^f^	33.5 ± 0.8
10	Cu(ClO_4_)_2_·6H_2_O^g^	42.1 ± 0.6^h^
11	CuCl_2_·2H_2_O	6.2 ± 0.8
12	LiClO_4_ ^d^ ^,^ ^e^	<1.0
13	CuCl_2_·2H_2_O + LiClO_4_	10.0 ± 1.1
14	CuCl_2_·2H_2_O + LiClO_4_ ^d^	10.1 ± 0.7
15	CuCl_2_·2H_2_O + LiClO_4_ ^e^	11.0 ± 1.3
16	Cu(NO_3_)_2_·3H_2_O	4.4 ± 0.7
17	Cu(NO_3_)_2_·3H_2_O + LiClO_4_ ^d^	10.1 ± 1.4
18	CuSO_4_·5H_2_O	<1.0
19	Cu(CH_3_COO)_2_·H_2_O	6.5 ± 0.5
20	Zn(ClO_4_)_2_·6H_2_O	1.2 ± 0.3
21		2.0 ± 1.3

a [Catalyst] = 7 × 10^–4^ mol L^–1^, *T* = 25 °C.

b Determined by GC-FID.

c [Cu] = 3.5 × 10^–3^ mol L^–1^.

d [ClO_4_^–^] = 3.5 × 10^–3^ mol L^–1^.

e [ClO_4_^–^] = 7 × 10^–3^ mol L^–1^.

f = reaction at 50 °C.

g = reaction at 75 °C.

h Determined by ^1^H NMR. Solvent: CH_3_CN. Time: 24 h. Only chlorocyclohexane was observed using GC-FID and ^1^H NMR analyses.

Given the superior result obtained with Cu­(ClO_4_)_2_·6H_2_O, other copper­(II) salts (Cl^–^, NO_3_^–^, and SO_4_^2–^) were evaluated. Interestingly, the conversions achieved with these salts were lower than those with the perchlorate salts (Table 1). This suggests that the perchlorate anion may play a significant role in the catalytic process. Therefore, a study was performed by adding LiClO_4_ to the system containing CuCl_2_·2H_2_O and Cu­(NO_3_)_2_·3H_2_O. The data (entries 15 and 17) revealed an increase in the catalytic activity after addition of LiClO_4_. Such an increase in the yield cannot be attributed to the Li^+^ cation since no conversion was observed in a control reaction containing only LiClO_4_ (entry 12). Dalpozzo and co-workers have reviewed the application of perchloric acid and their salts as catalysts in several organic reactions.^39^ The higher activity observed in several transition metal perchlorate salts has been attributed to the low Lewis basicity of the ClO_4_^–^ anion. This characteristic enables the metal center to function as a more effective Lewis acid when interacting with coordinating species, as ClO_4_^–^ is an uncoordinating anion. In contrast, Cl^–^, NO_3_^–^, and SO_4_^2–^ are Lewis bases stronger than ClO_4_^–^. Because these anions can coordinate with metal ions, they reduce the Lewis acidity of the copper center, leading to a decrease in activity.

### 3.2 Effect of the ClO_4_^–^ Concentration and Temperature on Conversion and Selectivity

After determining the importance of the Cu^2+^ and ClO_4_^–^ ions in the process, we attempted to enhance the conversion by increasing the concentration of ClO_4_^–^ in the solution through the addition of LiClO_4_. As shown in Table 1, entries 14 and 15, using ratios of 1:5 and 1:10 Cu­(II):ClO_4_, did not lead to an increase in yield, confirming the main role of the Cu­(II) ion and indicating that the ratio 1:2 of Cu:ClO_4_ is a satisfactory condition to reach good performance. Another approach to improve conversion involved increasing the Cu­(ClO_4_)_2_·6H_2_O concentration (entries 7 and 8), but no satisfactory increase was observed. On the other hand, the increase in the temperature (25–50 °C) had a substantial effect (entries 7 and 9) changing the conversion from 16 to 33% with approximately 100% selectivity to chlorocyclohexane. A study conducted at 75 °C (entry 10) yielded the highest amount of chlorocyclohexane, reaching 42%. However, this result came with decreased selectivity, as a small quantity of dichlorinated products was also detected. The identification of these dichlorinated products was only possible through the use of the GC-MS technique. No signals corresponding to such species were observed with ^1^H NMR or GC-FID analyses, which is likely due to the detection limits of these methods.

In this context, the study was expanded to evaluate some more complex substrates, such as *n*-hexane, cycloheptane, and norbornane, and was performed exclusively for the best catalytic system, consisting of copper­(II) perchlorate hexahydrate and TCCA. Mass spectrometry analyses of the reaction systems revealed that the major products were the monochlorinated compounds (Figures S2–S13). However, dichlorinated products were detected mainly at 75 °C, but as stated for cyclohexane, such products were not observed in ^1^H NMR analyses due to their low concentrations, falling below the detection limits of the NMR technique.

The quantification of the monochlorinated products in the chlorination reactions of the cited substrates with TCCA was measured using ^1^H NMR. Reactions were conducted with copper­(II) perchlorate hexahydrate as a catalyst and without the catalyst, at temperatures of 25, 50, and 75 °C for 24 h. The yields were determined based on the integral values of the substrate hydrogens, the monochlorinated products, and the internal standard, *N*,*N*-dimethylformamide. The study was performed in triplicate. The typical ^1^H NMR spectra are shown in the Supporting Information (Figures S14–S17).

In the reaction using cyclohexane at 25, 50, and 75 °C for 24 h, the highest yield was obtained at 75 °C, reaching approximately 42.1% (Figure 2).

**Figure 2:**
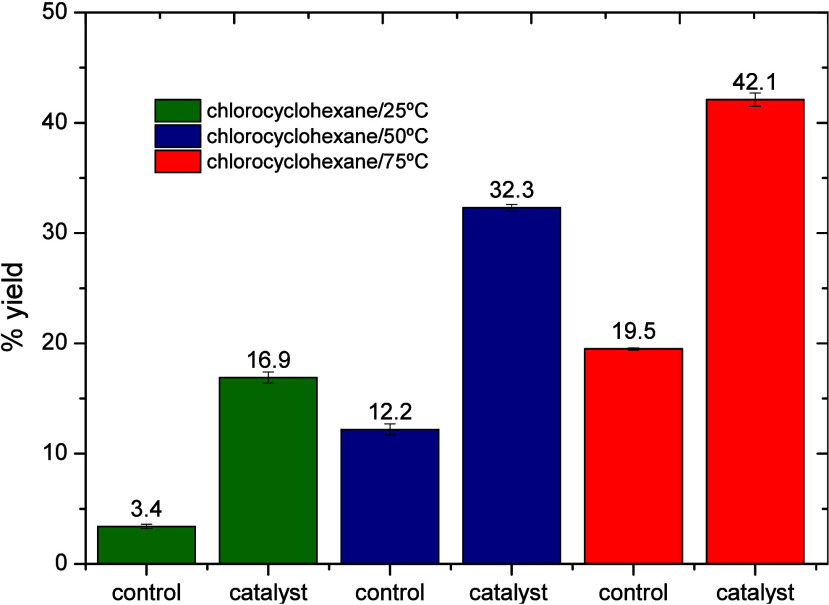
Yields of the product, chlorocyclohexane, at different temperatures, with and without the catalyst (Cu­(ClO_4_)_2_·6H_2_O).

Table 2 presents data related to the halogenation reactions promoted by copper, iron, and manganese complexes in the presence of TCCA, as described in the literature. Comparing the data at 50 °C for the copper compounds, it is possible to see that Cu­(ClO_4_)_2_.6H_2_O was more effective as a catalyst than the trinuclear complex [CuLCuLCu]­(ClO_4_)_2_,^38^ where H_2_L = 1,4-bis­(2-hydroxybenzyl)-1,4-diazepane. On the other hand, Cu­(ClO_4_)_2_.6H_2_O showed lower activity than the complex described by Melo and co-workers: [Cu­(BPAH)­(H_2_O)]­(ClO_4_)_2_.^27^ This illustrates that while the copper salt achieved good results in the formation of chlorocyclohexane, the development of copper coordination compounds can lead to improved catalytic systems. Additionally, the activity of these compounds can be fine-tuned by the type of ligand used in their synthesis. It is important to highly note that Cu­(ClO_4_)_2_.6H_2_O showed similar or better performance than the iron and manganese compounds.

**Table 2:** Comparison of Chlorocyclohexane Yields Using Different Coordination Compounds as Catalysts and TCCA as a Halogenating Agent, at 25 and 50 °C^a^

catalyst	temperature (°C)	yield (%)	reference
Cu(ClO_4_)_2_·6H_2_O	25	15.9	this work
	50	32.0	
	75	42.1	
[Cu(BPAH)(H_2_O)](ClO_4_)_2_	25	32.0	^27^
	50	44.7	
[CuLCuLCu](ClO_4_)_2_	25	8.1	^38^
	50	20.7	
[Fe(HBPCINOL)(Cl)_2_]	25	8.3	^26^
	50	34.4	
[Fe_2_(BPA)_2_(μ-OCH_3_)_2_(Cl)_2_]	25	7.9	^26^
	50	24.4	
[Mn(L^Met4^)Cl]	25	2.3	^28^
	50	15.1	
[Mn(salen)Cl]	25	14.5	^28^
	50	26.3	
[Mn(salan)Cl]	25	6.0	^28^
	50	19.4	

a BPAH = 1,4-bis­(propanamide)­homopiperazine; L = the dianion of the molecule 1,4-bis­(2-hydroxybenzyl)-1,4-diazepane; HBPCINOL = the monoanion of the molecule *N*-(2-hydroxybenzyl)-*N*-(2-pyridylmethyl)­(3-chloro)­(2-hydroxy)­propylamine; BPA = the monoanion of the molecule *N*-(2-hydroxybenzyl)-*N*-(pyridin-2-ylmethyl)­amine; L^Met4^ = the dianion of the molecule 6,6′-((1,4-diazepane-1,4-diyl)­bis­(methylene))­bis­(2,4-dimethylphenol); salen = the dianion of the molecule 2,2′-((1*E*,1′*E*)-(ethane-1,2-diylbis­(azaneylylidene))­bis­(methaneylylidene))­diphenol; salan = the dianion of the molecule 2,2′-((ethane-1,2-diylbis­(azanediyl))­bis­(methylene))­diphenol.

Due to the high conversion and selectivity for the monochlorinated product demonstrated by the Cu­(ClO_4_)_2_.6H_2_O system on cyclohexane, experiments were conducted with other substrates, *n*-hexane, cycloheptane, and norbornane that differ in carbon chain structure, number of primary, secondary, and tertiary carbons, stability of possible ions or radicals, intermediate conformations, and bond energies. The reaction with *n*-hexane (Table 3) resulted in the formation of the isomers 1-chlorohexane, 2-chlorohexane, and 3-chlorohexane (Figure 3), with total yields of 16.5% at 25 °C, 24.4% at 50 °C, and 41.1% at 75 °C in the catalyzed reactions. Although there is a 43% probability of the reaction occurring at the primary carbon atom and about 57% at the secondary carbon atoms (C2 = C3 ∼28%), the actual results did not reflect this ratio. The reactions predominantly took place at C2, yielding approximately 55% of 2-chlorohexane. In contrast, substitution at C1 was much lower than statistical predictions, yielding about 15, 14, and 8% at temperatures of 75, 50, and 25 °C, respectively. Over this temperature range, the percentage of products formed from reactions at C3 was between 30 and 36%. The ratio of products halogenated at C1:C2:C3 was close to 1:4:2 at 50 and 75 °C, changing to 1:6.5:4.3 at 25 °C. Such data are in contrast to the reported by Combe and co-workers, who found a ratio of 1:3:3 using a system typically involving radical species formed by TCCA, *N*-hydroxyphthalimide, and Cu­(OAc)_2_.^40^ This difference in selectivity may suggest that Cu­(ClO_4_)_2_.6H_2_O operates with a different mechanism. On the other hand, the data reported for the compound [Cu­(BPAH)­(H_2_O)]^2+^ were 1:4.8:3.^27^ The higher relative selectivity was found at 25 °C, 1­(C1):19.6­(C2):13.7­(C3).^27^

**Table 3:** Yields Obtained from the Chlorination Reactions of Cyclohexane, *n*-Hexane, Cycloheptane, and Norbornane, with TCCA, Catalyzed by Cu­(ClO_4_)_2_·6H_2_O (*1*), at 25, 50, and 75 °C for 24 h^a^

			yield (%)						
system	substrate	temperature	Cl-cyclohex	1-Cl-hex	2-Cl-hex	3-Cl-hex	Cl-cyclohep	*exo*-2-Cl-norb	total yield (%)
TCCA + 1	cyclohexane	75 °C	42.1 ± 0.6						42.1
TCCA	cyclohexane	75 °C	19.5 ± 0.1						19.5
TCCA + 1	cyclohexane	50 °C	32.3 ± 0.3						32.3
TCCA	cyclohexane	50 °C	12.2 ± 0.5						12.2
TCCA + 1	cyclohexane	25 °C	16.9 ± 0.5						16.9
TCCA	cyclohexane	25 °C	3.4 ± 0.2						3.4
TCCA + 1	*n*-hexane	75 °C		6.0 ± 0.3	22.7 ± 0.6	12.4 ± 0.6			41.1
TCCA	*n*-hexane	75 °C		1.7 ± 0.1	8.3 ± 0.7	4.9 ± 0.3			14.9
TCCA + 1	*n*-hexane	50 °C		3.4 ± 0.3	13.5 ± 0.5	7.5 ± 0.5			24.4
TCCA	*n*-hexane	50 °C		<1	3.4 ± 0.4	2.1 ± 0.1			∼5.5
TCCA + 1	*n*-hexane	25 °C		1.4 ± 0.2	9.1 ± 0.4	6.0 ± 0.7			16.5
TCCA	*n*-hexane	25 °C		<1	2.1 ± 0.1	1.4 ± 0.3			∼3.5
TCCA + 1	cycloheptane	75 °C					44.6 ± 0.7		44.6
TCCA	cycloheptane	75 °C					22.2 ± 0.1		22.2
TCCA + 1	cycloheptane	50 °C					32.0 ± 0.8		32.0
TCCA	cycloheptane	50 °C					14.2 ± 0.3		14.2
TCCA + 1	cycloheptane	25 °C					18.1 ± 0.2		18.1
TCCA	cycloheptane	25 °C					4.5 ± 0.4		4.5
TCCA + 1	norbornane	75 °C						42.1 ± 0.8	42.1
TCCA	norbornane	75 °C						20.5 ± 0.6	20.5
TCCA + 1	norbornane	50 °C						36.1 ± 0.1	36.1
TCCA	norbornane	50 °C						6.9 ± 0.3	6.9
TCCA + 1	norbornane	25 °C						16.0 ± 0.1	16.0
TCCA	norbornane	25 °C						4.0 ± 0.3	4.0

a 1 = Cu­(ClO_4_)_2_·6H_2_O; Cl-cyclohex = chlorocyclohexane; 1-Cl-hex = 1-chlorohexane; 2-Cl-hex = 2-chlorohexane; 3-Cl-hex = 3-chlorohexano; Cl-cyclohep = chlorocycloheptane; *exo*-2-Cl-norb = *exo*-2-chloronorbornane.

**Figure 3:**
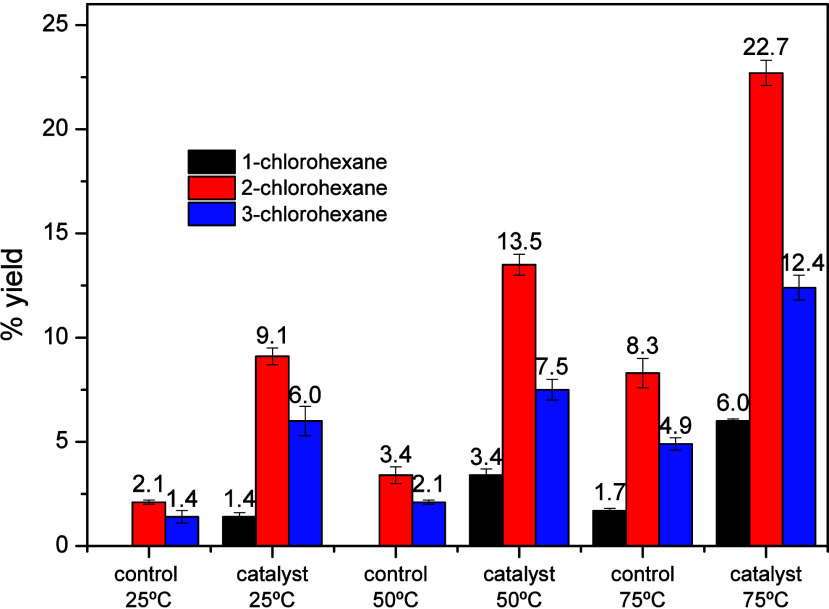
Yields of the products 1-chlorohexane, 2-chlorohexane, and 3-chlorohexane, at different temperatures, with and without the catalyst (Cu­(ClO_4_)_2_·6H_2_O).

Further than the statistical disponibility of hydrogen atoms on the secondary and primary carbon atoms, another factor that must be considered when evaluating the reactivity of the substrate is the bond energy dissociation (BDE). According to the theoretical calculations reported by Zheng and co-workers,^41^ the BDEs associated with the C–H bond in *n*-hexane are 101.3, 98.7, and 98.9 kcal mol^–1^ for C1, C2, and C3, respectively. Considering that the bond dissociation energies (BDE) for C2 and C3 are similar, these parameters do not account for the higher yield observed in the production of 2-chlorohexane compared to 3-chlorohexane. However, Olmos and co-workers have demonstrated that less hindered secondary carbon atoms exhibit greater reactivity.^42^ Their study indicated that C2 in *n*-hexane shows higher reactivity than C3, a finding that was also observed in our study.

The capability of Cu­(ClO_4_)_2_·6H_2_O in promoting alkane functionalization was also evaluated on cycloheptane. The achieved yields of the product, chlorocycloheptane, at 25, 50, and 75 °C for 24 h revealed that the maximum yield was obtained at 75 °C, reaching approximately 44.6% (Table 3 and Figure 4).

**Figure 4:**
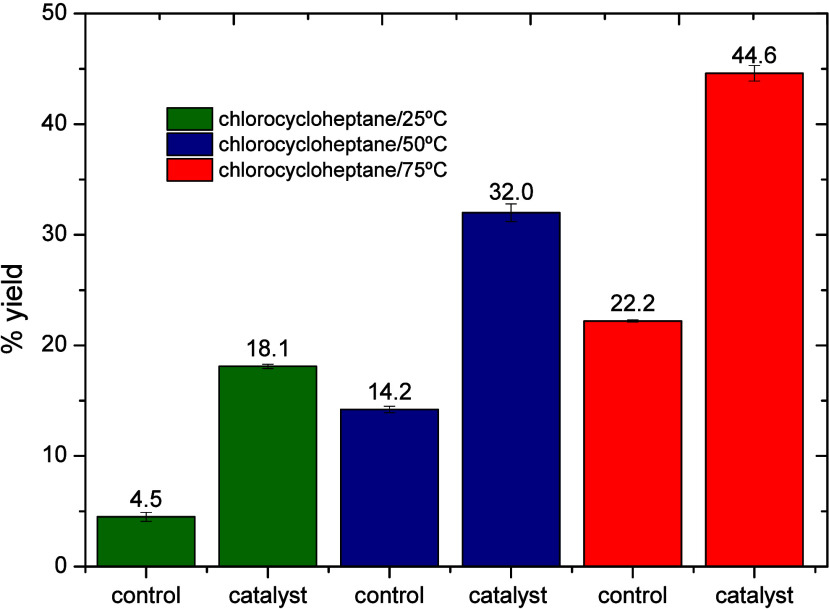
Yields of the product, chlorocycloheptane, at different temperatures, with and without the catalyst (Cu­(ClO_4_)_2_·6H_2_O).

The catalytic system was also evaluated employing norbornane as the substrate. The predominant formation of the *exo* isomer over the *endo* isomer was observed. Thus, the formation of exo-2-chloronorbornane reached a maximum yield of 42.1% at 75 °C (Table 3 and Figure 5).

**Figure 5:**
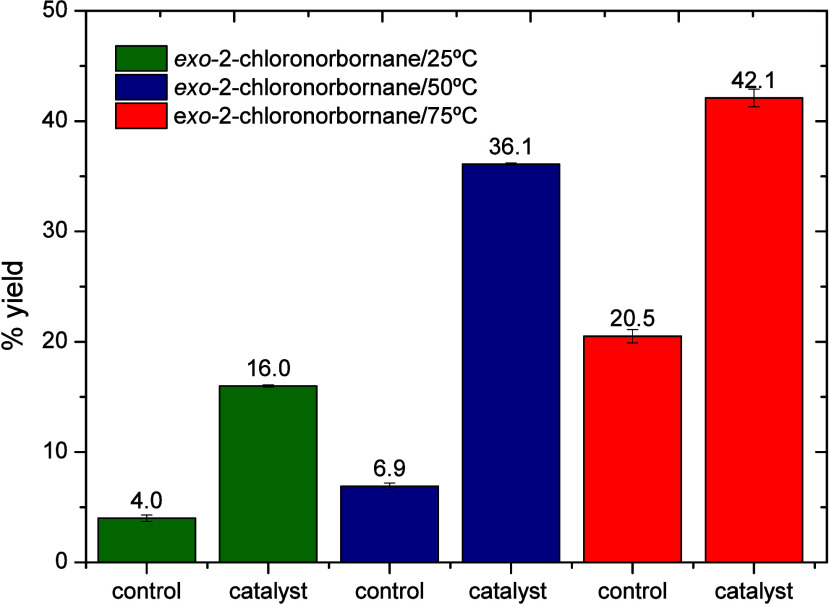
Yields of the product, exo-2-chloronorbornane, at different temperatures, with and without the catalyst (Cu­(ClO_4_)_2_·6H_2_O).

The catalytic efficiency of copper­(II) perchlorate hexahydrate over the different temperatures and substrates was compared with the yields obtained in reactions in the absence of the catalyst (Figure 6).

**Figure 6:**
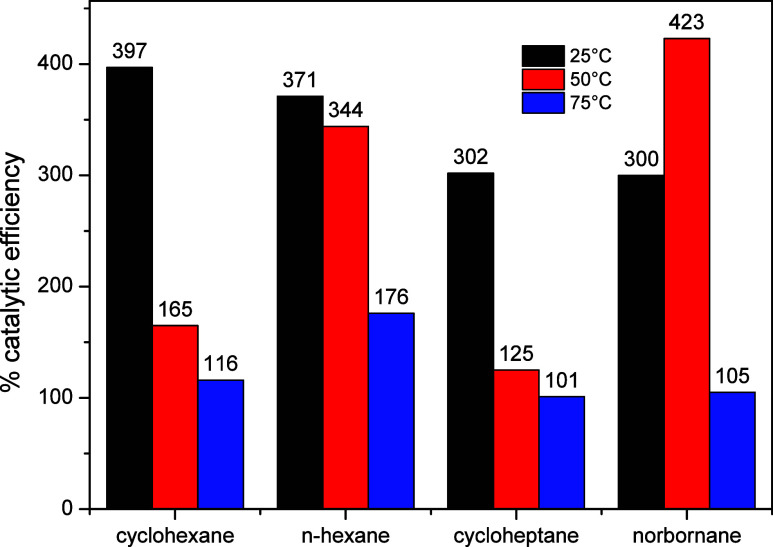
Catalytic efficiency of Cu­(ClO_4_)_2_·6H_2_O calculated based on the yields obtained from the chlorination reactions of all tested substrates, performed in the presence and in the absence of the catalyst. The catalytic efficiency was obtained by the ratio between the catalyzed and uncatalyzed reaction.

A comparison of the catalyzed reactions at various temperatures with the control reactions (Figure 6) clearly shows that the contribution of Cu­(ClO_4_)_2_·6H_2_O diminishes as the temperature rises with the exception of norbornane. The control reactions, which consist solely of the substrate and TCCA, exhibit higher conversion at elevated temperatures. This phenomenon has been observed in previous studies.^26–28,38^ Watkins and colleagues have suggested that the increase in temperature facilitates the formation of chlorine and nitrogen-centered radical species within the TCCA molecule, which explains the higher formation of chlorinated products at elevated temperatures.^43^

### 3.3 EPR Studies and Mechanistic Insights

According to the literature, the main mechanisms by which TCCA can promote the halogenation of organic molecules include (i) homolytic cleavage of the N–Cl bond, leading to the formation of N^•^ and Cl^•^ radicals;^33,44,45^ (ii) the heterolytic cleavage of the N–Cl bond, producing N^–^ and Cl^+^ species;^32,46^ and (iii) the generation of hypochlorite, which is activated by a metal (M) center in the reaction: M^*n*+^ + OCl^–^ → M–O^•^/M = O + Cl^•^.^26,27,38^ It has been proposed that both the N^•^ and M–O^•^/M = O species can abstract an H^•^ species from the substrate (R-H), resulting in the formation of an alkyl radical (R^•^). This process then leads to a reaction with Cl^•^, resulting in the formation of R-Cl species. Regarding the formation of Cl^+^, it has been proposed that it participates in reactions that involve an electrophilic attack, as exemplified in reactions with unsaturated hydrocarbons.

To better understand the higher activity of Cu­(ClO_4_)_2_ compared to other copper salts, EPR spectroscopy studies were conducted. Initially, the behavior of CuCl_2_ and Cu­(ClO_4_)_2_ in the presence of TCCA was investigated. At room temperature, both salts exhibited EPR isotropic signals typical of Cu­(II) ions in close proximity (Figure 7A,B). However, when TCCA was added, the EPR signal from the CuCl_2_ solution disappeared (Figure 7A), indicating that the Cu­(II) ion may have been reduced to Cu­(I) or oxidized to Cu­(III). In contrast, adding TCCA to the Cu­(ClO_4_)_2_ solution did not cause any significant change in the spectrum (Figure 7B). These observations confirm that the two copper salts interact with TCCA in different ways.

**Figure 7:**
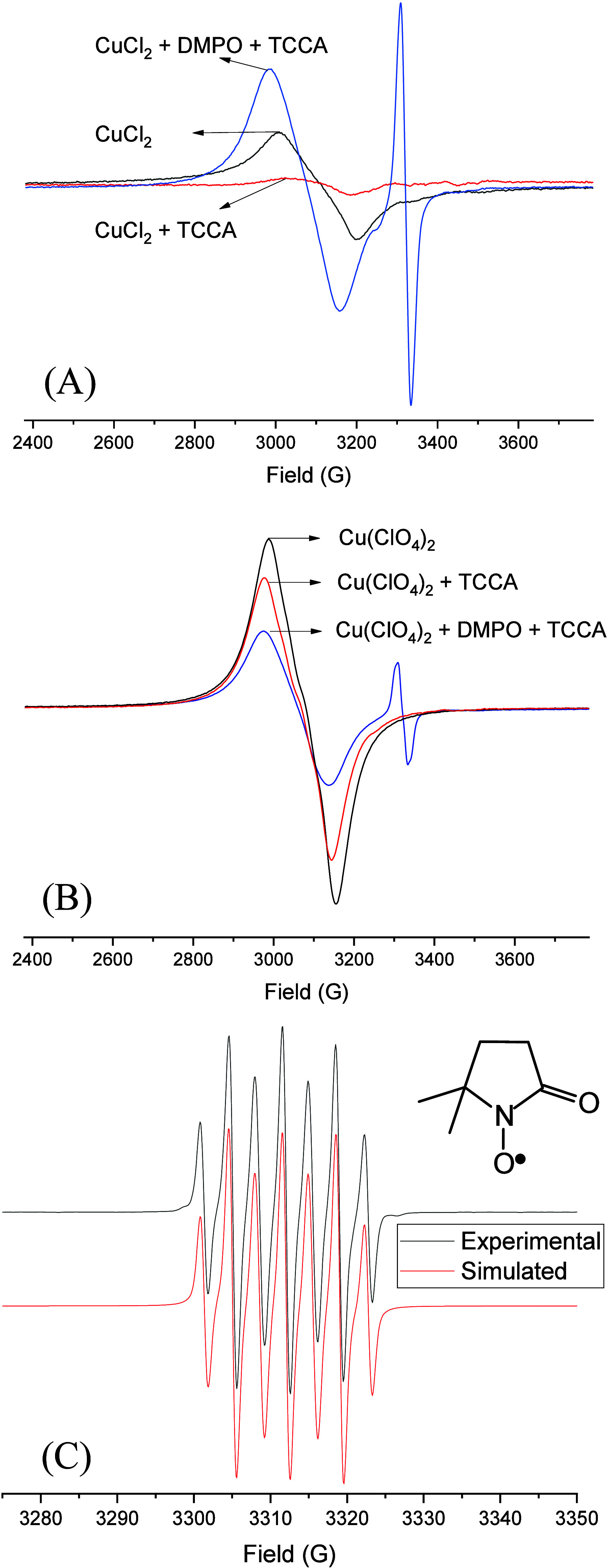
EPR spectra of acetonitrile solutions containing pure CuCl_2_ (A) and Cu­(ClO_4_)_2_ (B), as well as in the presence of TCCA and DMPO+TCCA, as indicated. (C) Experimental and simulated spectra of acetonitrile solution containing DMPO+TCCA. The structure shown in panel (C) illustrates the DMPOX species generated in the reaction between DMPO+TCCA, as indicated by spectral simulation *T* = 295 K.

A second study was conducted using the spin-trapping agent DMPO, which can trap radical species and stabilize nitrone radical adducts detectable by EPR. In the presence of the spin trap DMPO, the behavior of the solution containing CuCl_2_ changed significantly compared to the solution without DMPO (Figure 7A). As previously noted, the absence of DMPO resulted in a complete reduction of the characteristic signal associated with Cu­(II) in the presence of TCCA, suggesting a loss of detectable copper­(II) species. In contrast, an increase in the Cu­(II) signal was observed in the presence of DMPO, along with the emergence of a new signal around *g* ∼2.0, which is characteristic of a DMPO-radical adduct. The continued presence of the Cu­(II) signal indicates that TCCA, or a species generated by TCCA, preferentially reacts with DMPO rather than the Cu­(II) ion. In the equivalent system containing Cu­(ClO_4_)_2_, the DMPO-radical adduct was also observed but with lower intensity. These results also suggest that the interaction between Cu­(II) and TCCA differs in reactions involving CuCl_2_ and Cu­(ClO_4_)_2_.

To determine whether Cu­(II) ions are responsible for generating the radical species observed at *g* ∼2.0 in the systems containing the copper salts, DMPO and TCCA, a similar study was conducted without Cu­(II) ions. As shown in Figure 7C, the formation of a nitrone radical adduct was also observed in the absence of Cu­(II). Unfortunately, it is not possible to compare the radical species formed in the presence and absence of Cu­(II) ions since only broad signals were detected in the presence of Cu­(II) salts (Figure 7A,B), while in the absence of Cu­(II), a signal with seven distinct lines was observed (see Figure 7C). The simulation of such spectrum showed *a*_N_ = 6.96 G and a_H_^γ^(2H) = 3.76 G. In a study of the reaction between DMPO and ClO^–^, Bernofsky and co-workers have attributed such species to the oxidation of DMPO, forming a 5,5-dimethyl-2-pyrrolidone-1-oxyl radical (DMPOX, Figure 7C), with *a*_N_ = 7.2 G and a_H_^γ^(2H) = 4.1 G.^47^ A similar spectrum was observed by Shiozawa, who investigated the reaction between NaClO+H_2_O_2_+DMPO,^48^ and Stan and co-workers,^49^ who identify the same pattern during the electrolysis of a NaCl solution. All of these studies suggest that DMPOX is formed by the oxidation of DMPO by hypochlorite. Furthermore, using fluorescence spectroscopy, we have shown that a solution of TCCA quenched the umbelliferon fluorescence, a behavior observed for solutions containing ClO^–^.^38^ Together, the data indicate that TCCA in CH_3_CN solution produces ClO^–^. In the presence of CuCl_2_, it promotes reduction or oxidation of the Cu­(II) center. On the contrary, with Cu­(ClO_4_)_2_, such a reaction does not occur.

Theoretical calculations and experimental data have demonstrated that ClO^–^ can coordinate to a Cu­(II) center, leading to the cleavage of the chloro-oxygen bond,^27,38^ This process results in the formation of Cu-oxyl and chloro radical species. The Cu-oxyl can abstract a hydrogen radical from the substrate, while the chloro radical can react with the organic radical generated, resulting in the formation of a haloalkane molecule. Considering the experimental data and proposals reported in the literature, our hypothesis for the reaction mechanism promoted by Cu­(ClO_4_)_2_ involves the following steps:

$$\mathrm{TCCA}+H_{2}O\to 3\mathrm{HClO}+\mathrm{cyanuric}\;\mathrm{acid}$$ 1

$$\mathrm{HClO}\;↔\;H^{+}\;+\;{\mathrm{ClO}}^{-}$$ 2

$$\mathrm{Cu}(\mathrm{II})+{\mathrm{ClO}}^{-}\;\to \;\mathrm{Cu}-O-\mathrm{Cl}$$ 3

$$\mathrm{Cu}-O-\mathrm{Cl}\;\to \;\mathrm{Cu}-O^{•}\;+\;{\mathrm{Cl}}^{•}$$ 4

$$\mathrm{Cu}-O^{•}\;+\;R-H\;\to \;\mathrm{Cu}-\mathrm{OH}\;+\;R^{•}$$ 5

$$R^{•}\;+\;{\mathrm{Cl}}^{•}\;\to \;R-\mathrm{Cl}$$ 6

$$\mathrm{Cu}-\mathrm{OH}\;+\;H^{+}\;\to \;\mathrm{Cu}(\mathrm{II})\;+\;H_{2}O$$ 7

In the first step, hypochlorous acid (HClO) is generated through the hydrolysis of trichloroisocyanuric acid (TCCA) and exists in equilibrium with the hypochlorite ion (ClO^–^). In step 3, ClO^–^ interacts with the Cu­(II) ion, leading to the homolytic cleavage of the Cl–O bond (see eq 4). The resulting oxyl species, which is bound to the Cu­(II) center, abstracts a hydrogen atom from the substrate (eq 5), creating an alkyl radical. This alkyl radical then reacts with the chlorine radical species formed in eq 4, resulting in the formation of a haloalkane (eq 6). At the last step, water is formed by the protonation of the Cu–OH by the proton originating from HClO. It is important to note that this process occurs in a solution containing Cu­(ClO_4_)_2_, which accounts for the higher activity observed in this system. In contrast, in a solution containing CuCl_2_, TCCA interacts differently with the copper center (as discussed in the EPR section), which explains the lower activity exhibited by this system.

## 4 Conclusions

The investigation into the catalytic activity of various first-row transition metal salts in the chlorination reaction of cyclohexane with TCCA revealed that copper­(II) perchlorate hexahydrate emerged as the most effective catalyst. It demonstrated high selectivity for the monochlorinated species, particularly at temperatures of 25 and 50 °C. Studies involving cyclohexane, *n*-hexane, cycloheptane, and norbornane indicated that the highest conversion rates (42–45%) occurred at 75 °C. The presence of both Cu^2+^ and ClO_4_^–^ ions is crucial for the halogenation process since salts such as CuCl_2_, Cu­(NO_3_)_2_, CuSO_4_, and Cu­(OAc)_2_ exhibited significantly lower activity compared to Cu­(ClO_4_)_2_. Mechanistic investigations using EPR spectroscopy revealed that Cu­(ClO_4_)_2_ and CuCl_2_ react differently with TCCA. In the case of Cu­(ClO_4_)_2_, the isotropic signal of Cu­(II) remains, while it disappears for CuCl_2_, indicating that the latter is undergoing a redox reaction. Such a difference should impact the catalytic activity. Overall, the system utilizing Cu­(ClO_4_)_2_·6H_2_O and TCCA proved to be effective in the chlorination of cyclic, bicyclic, and acyclic alkanes under mild conditions. Its strong performance, ease of handling, and a low environmental impact make this methodology consistent with the principles of green chemistry.

## Supplementary Material



## Notes

GC-MS and ^1^H NMR data related to the catalytic reaction (PDF)
