# Supplementary material for: An Eco-Friendly Method for Saturated Hydrocarbon Chlorination: Exploring the Potential of First-Row Transition Metal Ions
Source: ACS Omega. 2025 Jun 25;10(26):27936–44. doi: 10.1021/acsomega.5c01390 (PMC12242647; doi:10.1021/acsomega.5c01390)
Supplement: Supplementary file 1 [file ao5c01390_si_001.pdf]

Supplementary Material Associated with the Manuscript

An eco-friendly method for saturated hydrocarbon chlorination: exploring the potential of first-row transition metal ions

Eduardo S. Neves,[a, c] Leonardo M. Lube,[b] Lívia R. Oliveira,[c] Christiane Fernandes,[c] and Adolfo Horn Jr.*[c]

[a] Laboratório de Ciências Químicas, Universidade Estadual do Norte Fluminense Darcy Ribeiro

Campos dos Goytacazes, 28013-602, RJ, Brazil

[b] Instituto Federal Fluminense, Campus Campos Centro, Campos dos Goytacazes, 28030130, RJ, Brazil

[c] Departamento de Química, Universidade Federal de Santa Catarina, Florianópolis, 88040-900, SC, Brazil

E-mail: adolfo.junior@ufsc.br

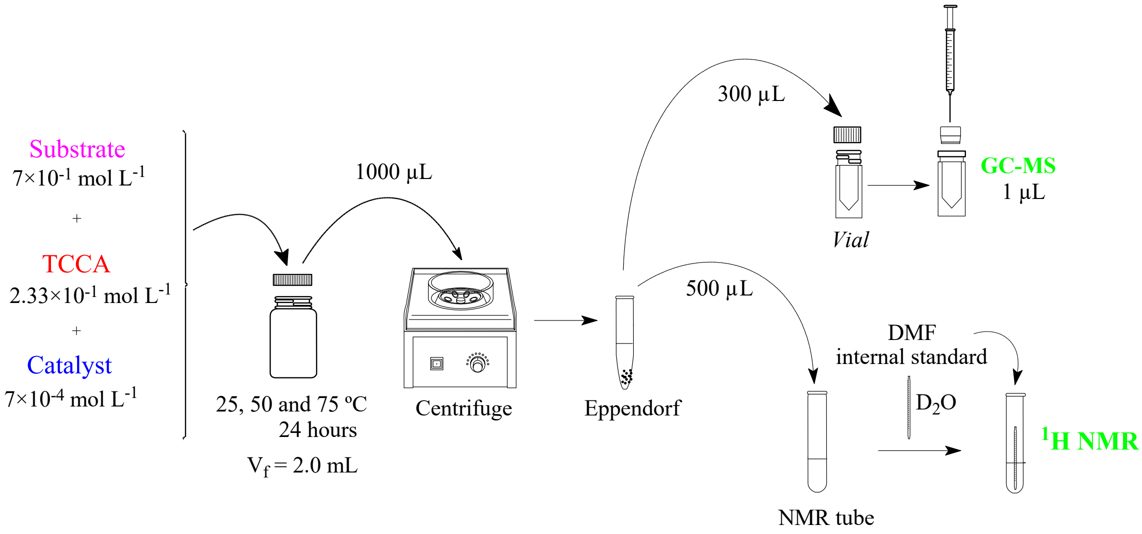

Figure S1. Experimental scheme showing the steps employed in the evaluation of the catalytic activity.

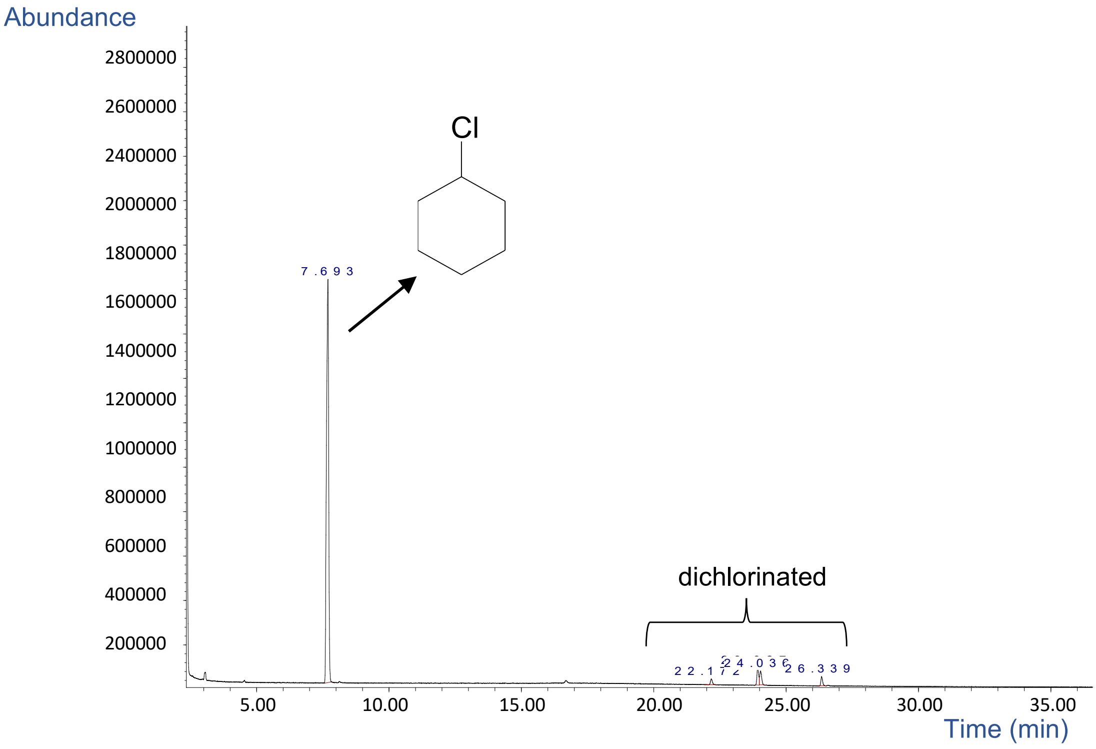

Figure S2. Chlorination reaction chromatogram of cyclohexane with TCCA and $Cu(ClO_4)_2 \cdot 6H_2O$ at 25 ºC

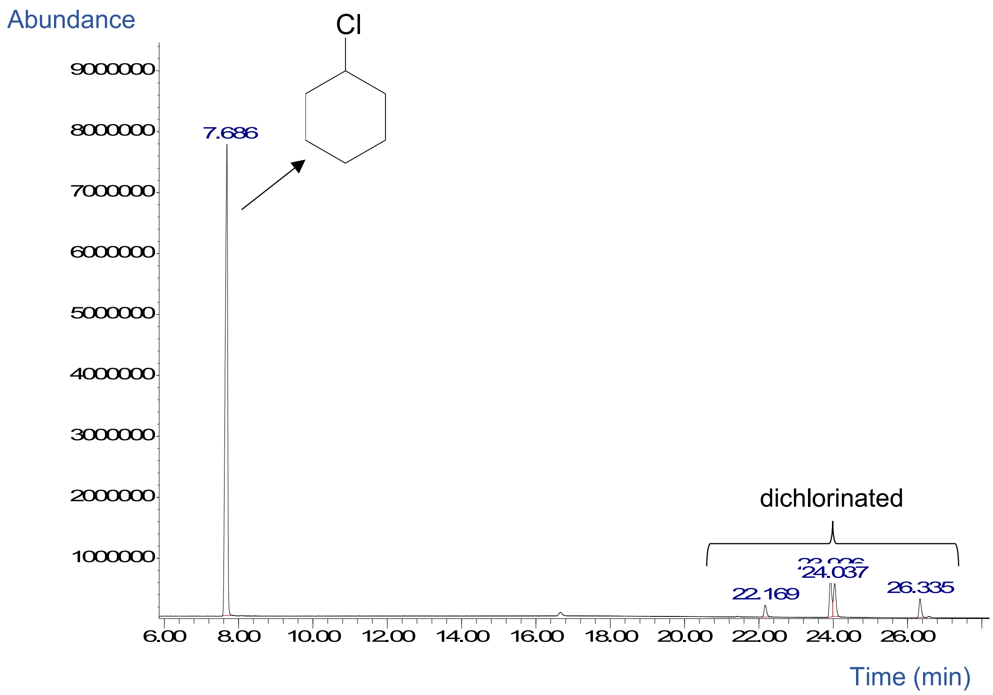

Figure S3. Chlorination reaction chromatogram of cyclohexane with TCCA and $Cu(ClO_4)_2 \cdot 6H_2O$ at 50 ºC.

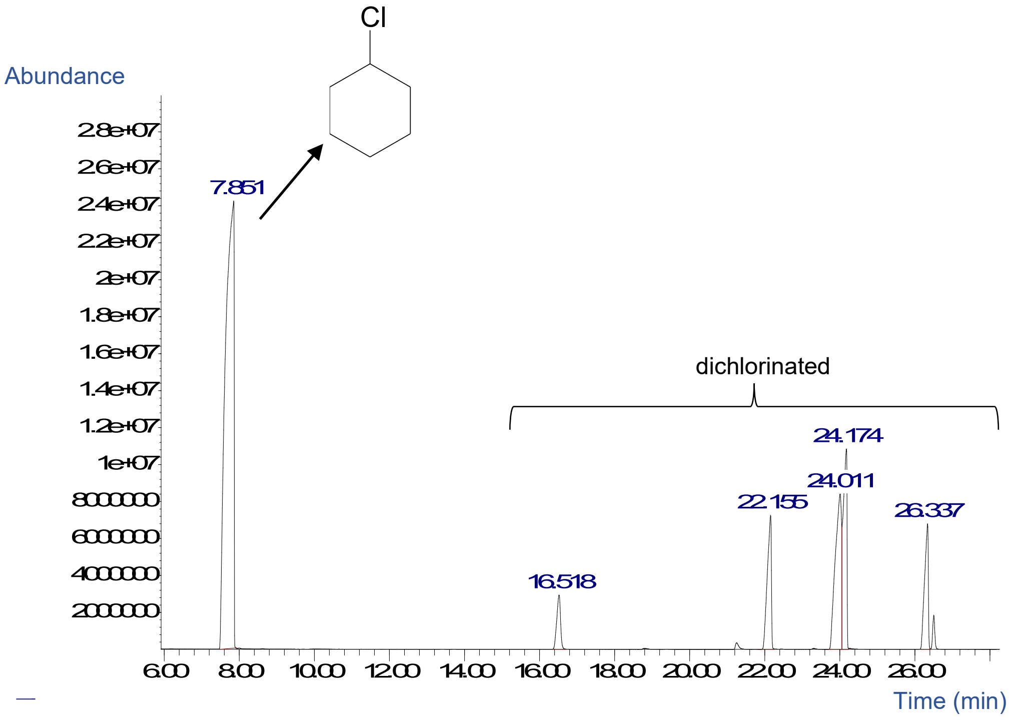

Figure S4. Chlorination reaction chromatogram of cyclohexane with TCCA and $Cu(ClO_4)_2 \cdot 6H_2O$ at 75 ºC.

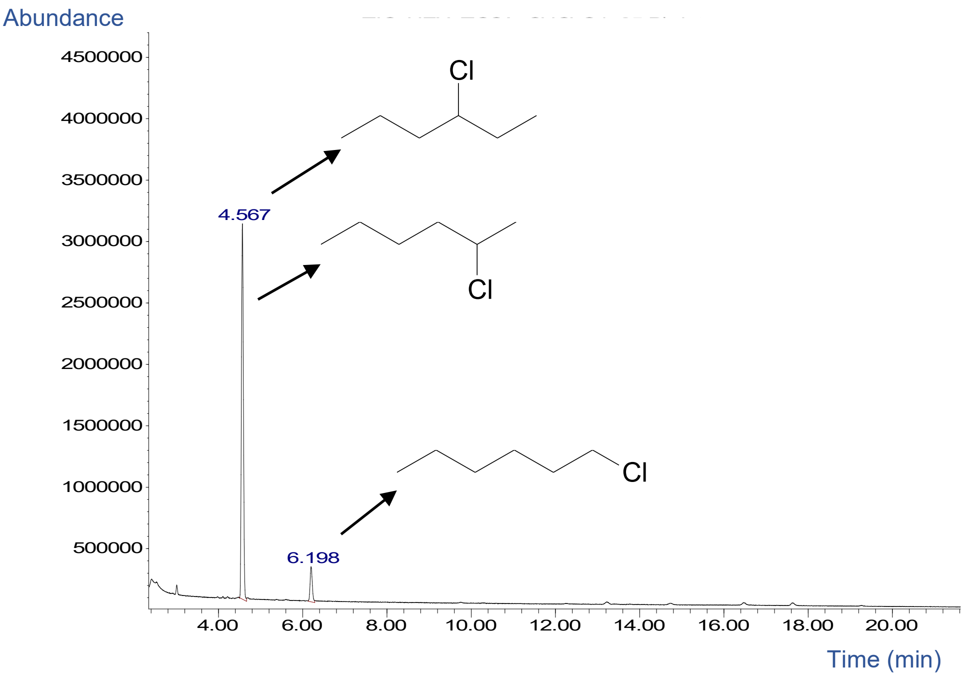

Figure S5. Chlorination reaction chromatogram of n-hexane with TCCA and $Cu(ClO_4)_2 \cdot 6H_2O$ at 25 ºC.

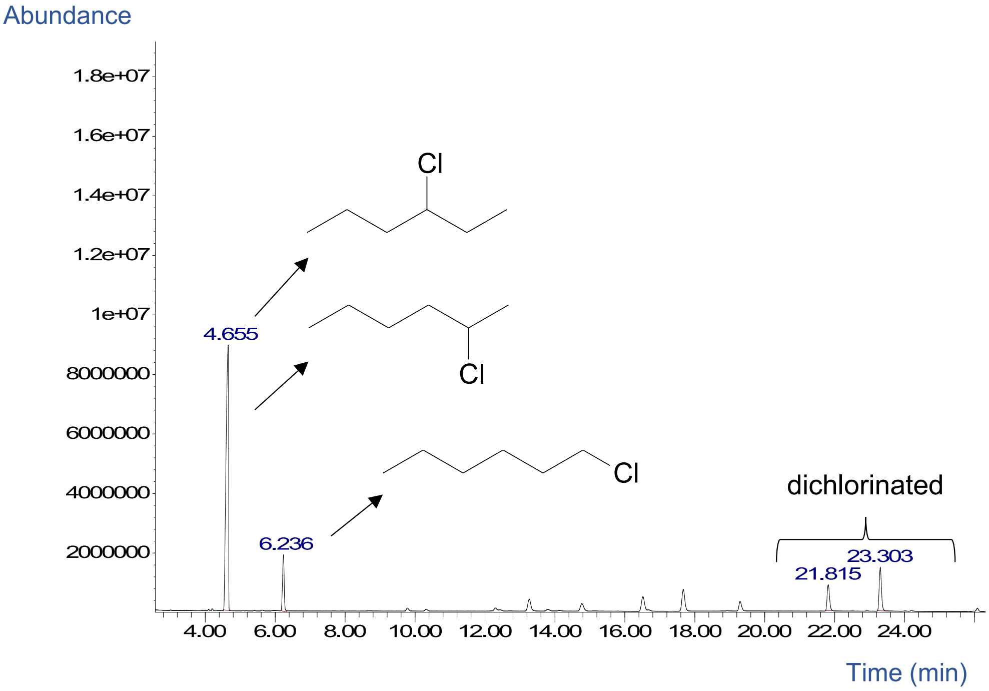

Figure S6. Chlorination reaction chromatogram of n-hexane with TCCA and $Cu(ClO_4)_2 \cdot 6H_2O$ at 50 ºC.

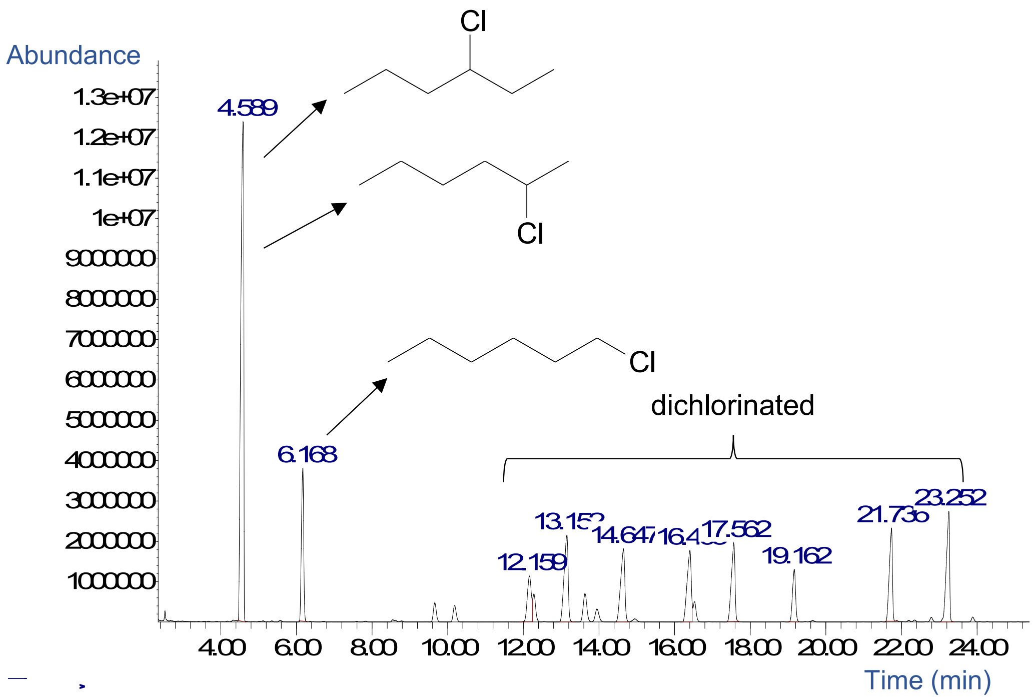

Figure S7. Chlorination reaction chromatogram of n-hexane with TCCA and $Cu(ClO_4)_2{\cdot}6H_2O$ at 75 ºC.

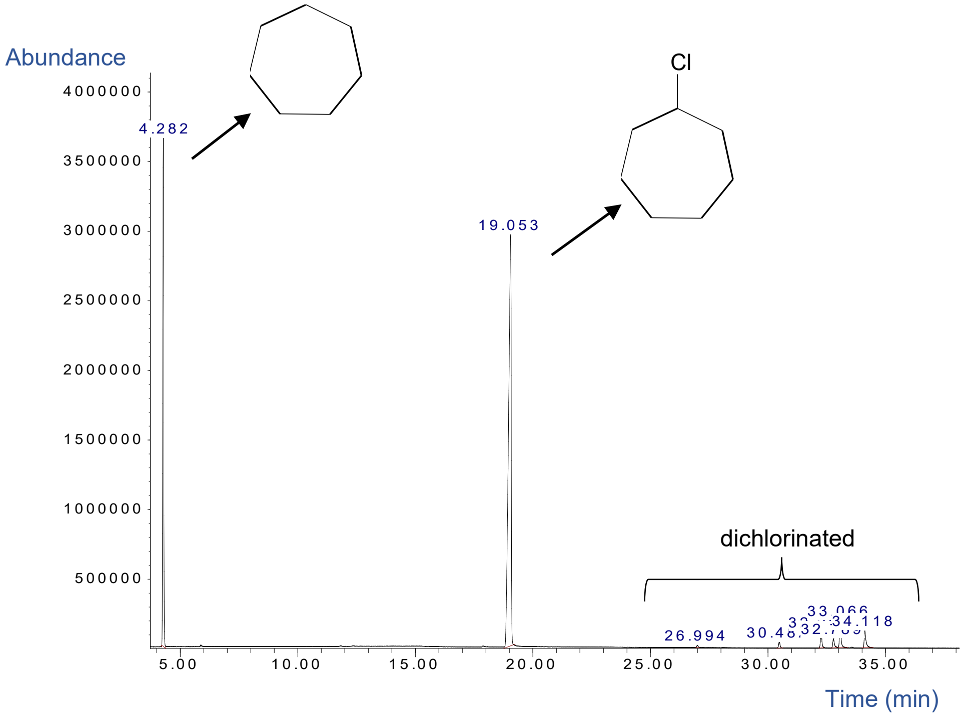

Figure S8. Chlorination reaction chromatogram of cycloheptane with TCCA and $Cu(ClO_4)_2{\cdot}6H_2O$ at 25 ºC.

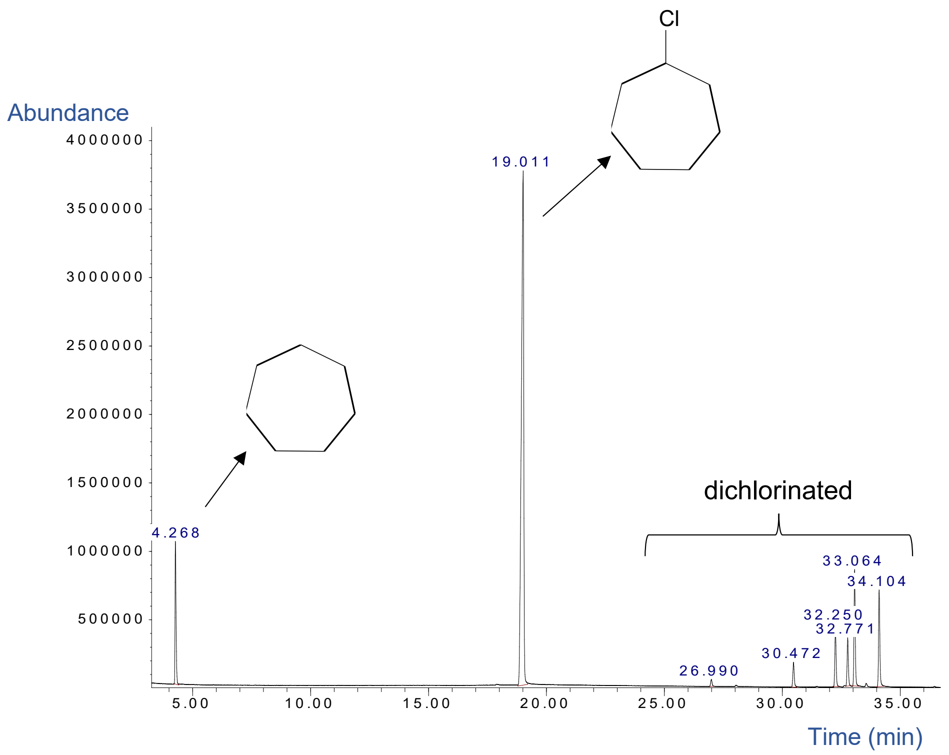

Figure S9. Chlorination reaction chromatogram of cycloheptane with TCCA and $Cu(ClO_4)_2{\cdot}6H_2O$ at 50 ºC.

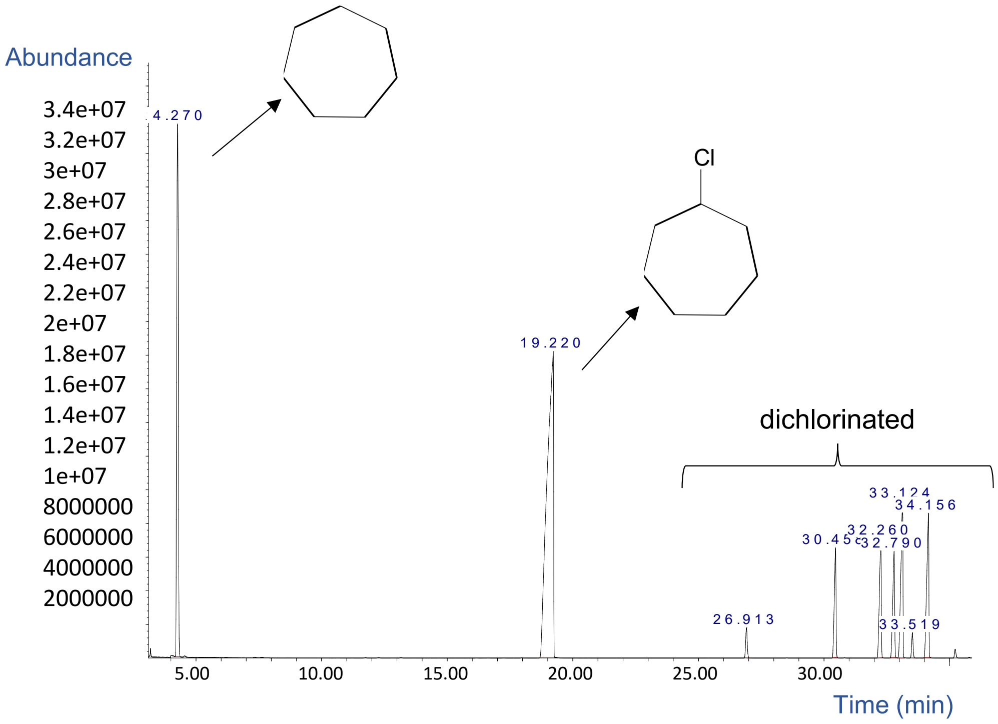

Figure S10. Chlorination reaction chromatogram of cycloheptane with TCCA and $Cu(ClO_4)_2{\cdot}6H_2O$ at 75 ºC.

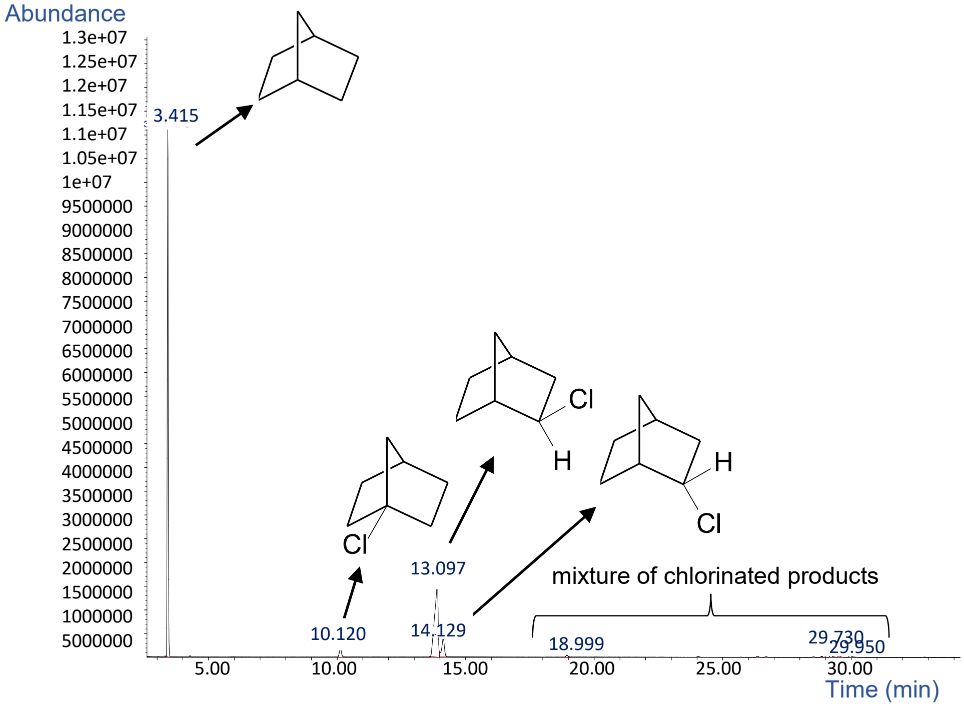

Figure S11. Chlorination reaction chromatogram of norbornane with TCCA and $Cu(ClO_4)_2{\cdot}6H_2O$ at 25 ºC.

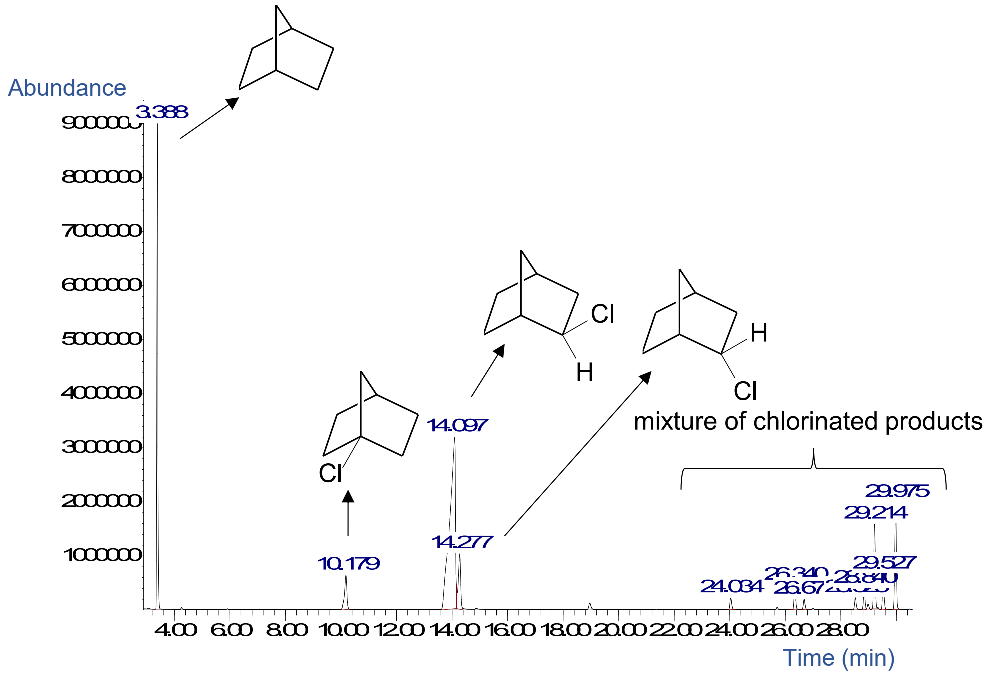

Figure S12. Chlorination reaction chromatogram of norbornane with TCCA and $Cu(ClO_4)_2{\cdot}6H_2O$ at 50 ºC.

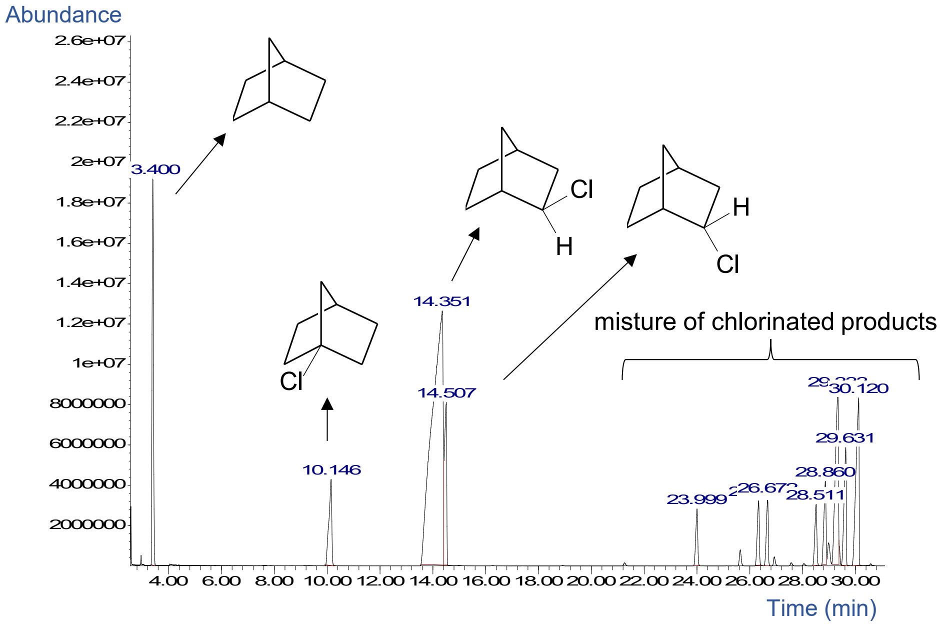

Figure S13. Chlorination reaction chromatogram of norbornane with TCCA and $Cu(ClO_4)_2{\cdot}6H_2O$ at 75 ºC.

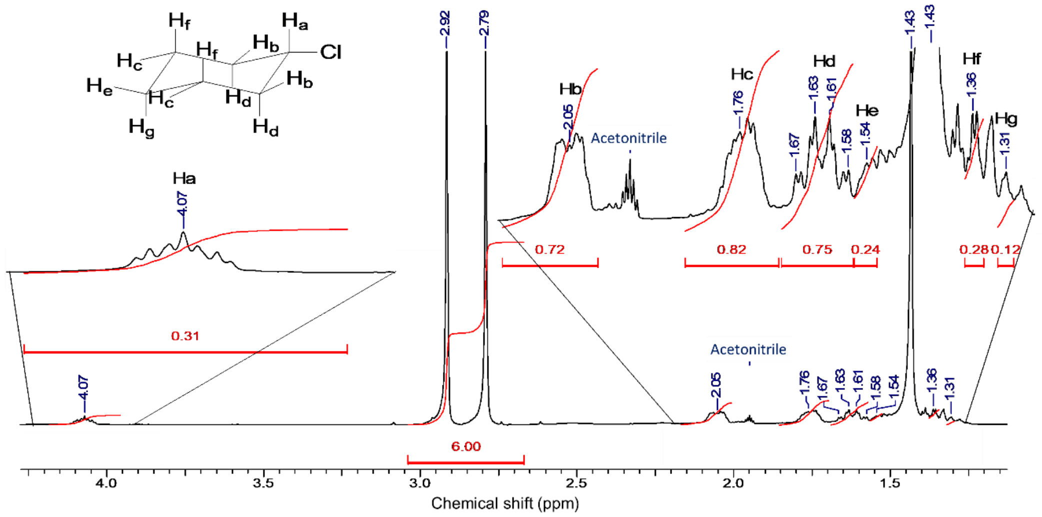

Figure S14. ^{1}H NMR spectrum of the reaction of cyclohexane with TCCA catalyzed by $Cu(ClO_4)_2{\cdot}6H_2O$ at 50 ºC, obtained in CD_3CN operating at 400 MHz for [1]H.

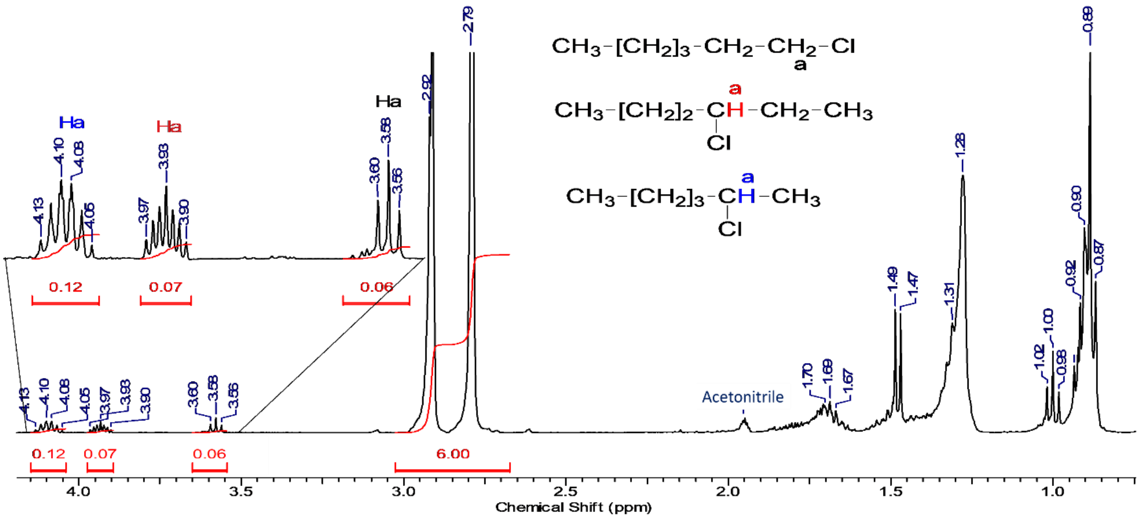

Figure S15. ^{1}H NMR spectrum of the reaction of n-hexane with TCCA catalyzed by $Cu(ClO_4)_2 \cdot 6H_2O$ at 50 ºC, obtained in CD_3CN operating at 400 MHz for ^{1}H.

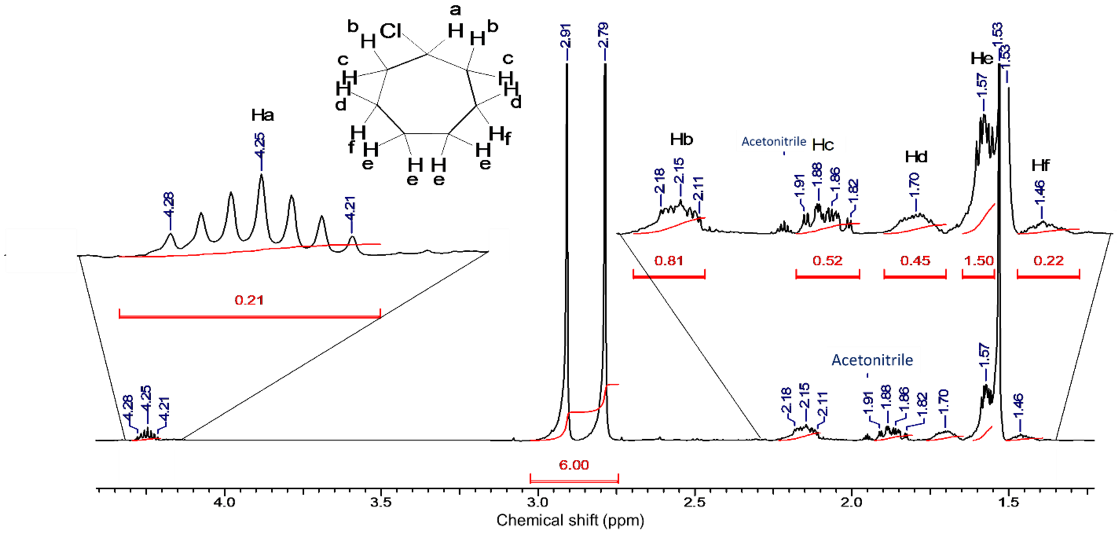

Figure S16. ^{1}H NMR spectrum of the reaction of cycloheptane with TCCA catalyzed by $Cu(ClO_4)_2 \cdot 6H_2O$ at 50 ºC, obtained in CD_3CN operating at 400 MHz for ^{1}H.

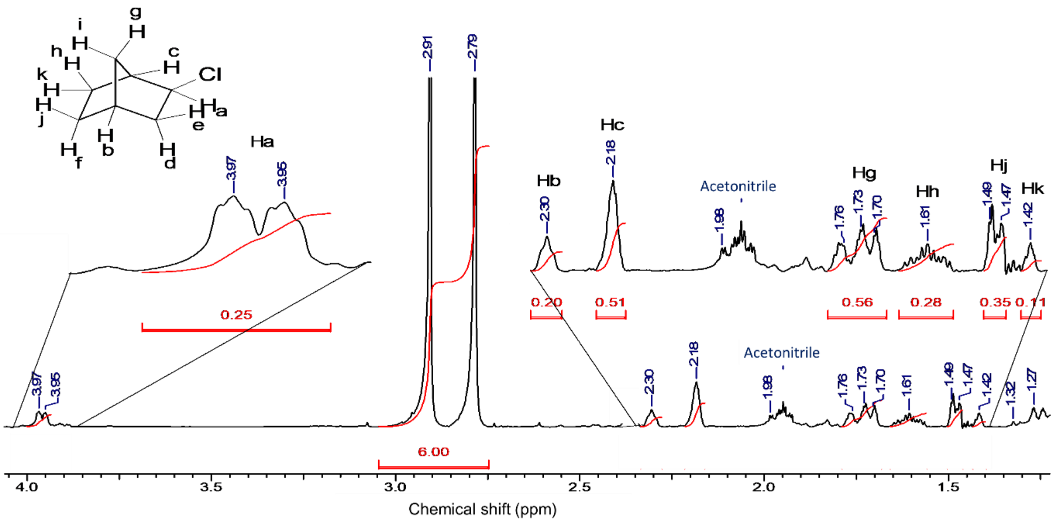

Figure S17. ^{1}H NMR spectrum of the reaction of cyclohexane with TCCA catalyzed by $Cu(ClO_4)_2 \cdot 6H_2O$ at 50 ºC, obtained in CD_3CN operating at 400 MHz for ^{1}H.
